# The effect of adherence to statin therapy on cardiovascular mortality: quantification of unmeasured bias using falsification end-points

**DOI:** 10.1186/s12889-016-2986-0

**Published:** 2016-04-11

**Authors:** Maarten J. Bijlsma, Stijn Vansteelandt, Fanny Janssen, Eelko Hak

**Affiliations:** Unit PharmacoEpidemiology & PharmacoEconomics (PE2), Department of Pharmacy, University of Groningen, A. Deusinglaan 1, PO BOX 9713 AV, Groningen, The Netherlands; Department of Applied Mathematics, Computer Science and Statistics, Ghent University, Krijgslaan 281, S9, 9000 Ghent, Belgium; Population Research Centre (PRC), Faculty of Spatial Sciences, University of Groningen, Landleven 1, 9747 AD Groningen, The Netherlands

**Keywords:** Statin therapy, Cardiovascular, Mortality, Bias, Overadjustment, Falsification end-points

## Abstract

**Background:**

To determine the clinical effectiveness of statins on cardiovascular mortality in practice, observational studies are needed. Control for confounding is essential in any observational study. Falsification end-points may be useful to determine if bias is present after adjustment has taken place.

**Methods:**

We followed starters on statin therapy in the Netherlands aged 46 to 100 years over the period 1996 to 2012, from initiation of statin therapy until cardiovascular mortality or censoring. Within this group (*n* = 49,688, up to 16 years of follow-up), we estimated the effect of adherence to statin therapy (0 = completely non-adherent, 1 = fully adherent) on ischemic heart diseases and cerebrovascular disease (ICD10-codes I20-I25 and I60-I69) as well as respiratory and endocrine disease mortality (ICD10-codes J00-J99 and E00-E90) as falsification end points, controlling for demographic factors, socio-economic factors, birth cohort, adherence to other cardiovascular medications, and diabetes using time-varying Cox regression models.

**Results:**

Falsification end-points indicated that a simpler model was less biased than a model with more controls. Adherence to statins appeared to be protective against cardiovascular mortality (HR: 0.70, 95 % CI 0.61 to 0.81).

**Conclusions:**

Falsification end-points helped detect overadjustment bias or bias due to competing risks, and thereby proved to be a useful technique in such a complex setting.

## Background

Statins are cholesterol lowering drugs whose efficacy in reducing cardiovascular mortality was demonstrated in various clinical trials (e.g. [1, 2]). However, evidence from trials does not necessarily give a good indication of drug effects for end users; trial participants differ from patients in clinical practice in terms of demography, concomitant drug use and co-morbidity [3, 4]. To determine the clinical effectiveness of drugs, observational studies are needed. However, in an observational setting, confounding factors may distort effect estimates. In observational studies of the effect of statin therapy on cardiovascular outcomes, healthy user bias and confounding by indication are most likely present, though many other sources of bias may also exist.

Patients who are prescribed statins have a higher baseline risk of cardiovascular mortality than patients who have not been prescribed statins. Therefore, comparisons of statin-users cannot easily be compared with non-users, risking confounding by indication. By comparing statin-users among each other, for example by looking at adherence to prescribed regimen, confounding by indication is reduced. However, such a comparison risks healthy adherer bias because higher adherence may correlate with a healthier lifestyle and higher adherence to other cardiovascular drugs. Ideally, such factors are controlled. In the absence of direct measures of lifestyle, behavioral proxies such as neighborhood characteristics or birth cohort may provide a solution [5–7].

The utility of proxies to reduce confounding is setting dependent, and may be unknown. Therefore, other checks are also required. In particular, falsification end-points (also known as negative controls) may provide a useful indicator of bias [8]. Falsification end-points are outcomes that are not causally affected by the primary exposure. If the primary exposure appears effective in reducing (or increasing) the risk of the primary outcome, this is an indication of bias, though the reverse is not necessarily true [9].

The aim of this study is to investigate the role of bias in an assessment of the effect of adherence to statin therapy on cardiovascular & cerebrovascular mortality among statin users in the Netherlands over the period 1994 to 2010.

## Methods

### Study population of starters of statin therapy

The study population consisted of outpatients that initiated statin therapy between ages 46 and 100 in the study period 1996 to 2012, belonging to birth cohorts 1911 to 1960 in the Netherlands. These age and time ranges constitute nearly all statin users in the Netherlands in the past decades; prevalence of statin use remains extremely below age 45 years and statins were introduced in the Netherlands around 1994 [5]. Approval from an institutional review board was not required to perform this study.

### Data sources

We linked outpatient pharmacy data from the University Groningen drug dispensing database (IADB.nl) to patient-level and neighborhood-level data from Statistics Netherlands. The IADB contains dispensing information from 55 community pharmacies in the Netherlands, covering on average 500,000 persons annually (www.IADB.nl) [10]. The database’s pharmacy information includes, among others, name of the drug, anatomic–therapeutic–chemical (ATC) classification and date of dispensing. With the exception of over-the-counter drugs and in-hospital dispensings, all dispensings are included regardless of prescriber, insurance, or reimbursement status. Medication records of patients are virtually complete because of high patient pharmacy commitment in the Netherlands [10]. The IADB ensures anonymity of patients by using anonymous identifiers. The IADB has been used in previous studies on statin use [5, 11]. IADB data was linked to data on socio-economic covariates from Statistics Netherlands using deterministic linkage based on date of birth, sex and location of residence at various points in time. For this study, we selected patients who were part of the catchment area of the IADB pharmacies, but were not living in areas from which patients were more likely to visit other (non-IADB) pharmacies. Patients could be followed up to 16 years. Patients that moved out of the IADB area were censored, as they are then more likely to receive drugs from other pharmacies.

### Data availability

Data from the IADB and Statistics Netherlands were not openly accessible. They were accessed after receiving permission from IADB (IADB.nl) and Statistics Netherlands, respectively. Linkage of the two datasets was performed by Statistics Netherlands, with permission and cooperation from the IADB.

### Primary exposure

The primary exposure of interest is time-varying adherence to statin therapy (ATC-code C10AA and C10B) [12]. In short, the time-varying adherence method calculates adherence using the proportion of days covered between dispensing *k* and dispensing *k + 2*, and then averages the adherence to 30-days intervals [12]. We included all starters of statin therapy in the database. Individuals were considered to be a starter of statin therapy if they did not receive statins in a period of 12 months prior to a statin dispensing. The first date of dispensing was considered the index date.

### Primary outcome measure

The outcome of this study is time from initiation of statin therapy to cardiovascular mortality in 30-day units. Cardiovascular mortality was defined as mortality due to ischemic heart diseases (ICD10-codes I20-I25) or mortality due to cerebrovascular disease (ICD10-codes I60-I69) [13].

### Falsification outcome measure

Statins should primarily reduce cardiovascular mortality through a reduction in blood lipid concentration. Therefore, it should not have a strong protective effect against mortality due to diseases of the respiratory system (ICD10-codes J0-J99) and endocrine, nutritional and metabolic diseases (ICD-10 code E0-99). The endocrine disorders include diseases of the thyroid, pituitary and adrenal gland, pancreas and gonads. We applied our small and large models also to these causes of deaths, which can therefore be seen as negative controls, also known as a falsification endpoint [14, 15].

### Patient-level covariates

Patient-level variables that were included in the modelling process, because they represented potential confounders, were demographic variables, drug utilization variables and calendar year of observation. The demographic variables were: sex (male or female), and age in 5-year categories (going from category 46–50 to category 96–100). Drug utilization variables were time-varying variables measuring adherence and exposure levels of the following drugs: drugs used in diabetes (ATC code A10), anti-inflammatory and anti-rheumatic drugs (ATC M01), anti-thrombotic drugs (ATC B01), drugs for obstructive airway diseases (ATC R03), cardiac therapeutics (ATC C01), anti-hypertensives (ATC C02), diuretics (ATC C03; this category also includes important anti-hypertensives), beta blocking agents (ATC C07), calcium channel blockers (ATC C08) and agents acting on the renin-angiotensin system (ATC C09). Drug exposure level was measured in daily defined dosage (DDD).

### Aggregate-level covariates

In the modelling process, we also included a variable on neighborhood socio-economic score (SES), and birth cohort in 5-year categories (going from category 1911–1915 until category 1956–1960). Both of these variables may be associated with health behavioral information [5–7, 16]. Adjustment for these variables may therefore reduce the influence of healthy adherer bias. Birth cohort has been shown to be associated both with drug utilization and with cardiovascular outcomes [5, 17–20]. Since the potential of birth cohort to confound or to modify effects is less known, we also specifically tested whether birth cohort contained confounding information by fitting models with and without birth cohort, and tested whether it was an effect modifier.

### Statistical analysis

To measure the effect of statin adherence on the hazard of cardiovascular mortality while controlling for other variables, we applied Cox models with time from initiation of statin therapy to cardiovascular mortality as the outcome. Patients who experienced mortality due to other causes of death were censored at their transition time. We lagged drug utilization variables by one year relative to the outcome as we did not expect changes in drug regimen to have a short-term effect on cardiovascular mortality.

Firstly, we built a model with statin adherence and statin drug exposure level, age, and calendar year. We used partial likelihood ratio tests to determine if any of these variables should be entered as categorical variables or as continuous variables (and potentially continuous with a squared term). We refer to this model as the ‘small’ model, due to many potential confounders being excluded from it. Secondly, we built the ‘large’ model using the following variable inclusion criteria: variables were added if they significantly added to the model according to a partial likelihood ratio test (first step), or if they did not significantly add to the model but their addition resulted in a more than 5 % change in the statin effect estimate (second step, as non-significant variables may also be confounders). Once the model was built, birth cohort was entered as a categorical variable. In order to avoid an identification problem occurring due to the linear dependency between age, period and cohort [21], we constrained the effect of the 1916–1920 birth cohort to be equal to that of the 1951–1955 cohort. Due to these constraints, birth cohort only measured non-linear effects. Statistical interaction terms between statin adherence and birth cohort were added to the model and a partial likelihood ratio test was used to determine the presence of effect modification.

Cox models are non-collapsible, i.e. conditional effect estimates of the model may not equal population-averaged effect estimates [22]. Therefore, to determine the public health effect of statin adherence, we also applied the parametric G-formula [23, 24]. This meant that we fitted regression models to our empirical data to estimate the complete joint distribution of cardiovascular mortality, censoring, and measured confounders. This estimated joint distribution was then used to simulate the risk of cardiovascular mortality if all patients were 100 % adherent, and to compare it with the simulated risk if all patients were 0 % adherent. This produced a population-averaged hazard ratio of cardiovascular mortality. Potential confounding by birth cohort was determined by comparing a hazard ratio produced in this manner while including birth cohort in the estimation of the joint distribution, with the hazard ratio produced without including birth cohort in the estimation.

### Subset analyses

We determined the influence of prescribing guidelines on the amount of measured confounding by applying the small Cox model and then the large Cox model for patients in the period 1996–2002, 2003–2006, and 2007–2012 separately, and then comparing the effect estimates.

## Results

### Patient information

The sample consisted of 49,688 patients, of which 52 % was male. The majority of patients at the start of statin therapy, and thereby at the start of follow up, were in age category 56–60 years. Of the patients that were censored, more than 90 % were censored in the final calendar year of study. The majority of patients also received other cardiovascular drugs next to statins (Table 1).

**Table 1:** Percentage of users in the sample with at least one dispensing for each drug

Drug type	ATC-code	% of patients with at least one dispensing
Antithrombotics	B01	61 %
Renin-angiotensin	C09	63 %
Beta blockers	C07	60 %
Diuretics	C03	56 %
Antidiabetics	A10	33 %
Anti-inflammatory	M01	34 %
Calcium channel blockers	C08	31 %
Cardiac therapy	C01	20 %
Obstructive airway	R03	19 %
Antihypertensives ^a^	C02	2 %

^a^the category ‘Diuretics’ includes important drugs used for antihypertensive properties

In our data, 64.8 % of patients started on Simvastatin, 19.1 % on Atorvastatin and 9.3 % on Pravastatin, with the remainder being other types of statins. However, some patients also switched drugs during follow-up. During follow-up, 32.7 % of patients switched to another statin or to a fibrate. Switchers switched on average 2.8 times. By far the most common switch was from Simvastatin to Atorvastatin (Table 2).

**Table 2:** Percentage of patients that switched cardiovascular drug therapy

Drugs switched	% of patients that switched
Simvastatin (C10AA01) to Atorvastatin (C10AA05)	12.0 %
Atorvastatin (C10AA05) to Simvastatin (C10AA01)	9.0 %
Gemfibrozil (C10AB04) to Simvastatin (C10AA01)	6.8 %
Simvastatin (C10AA01) to Gemfibrozil (C10AB04)	6.8 %
Simvastatin (C10AA01) to Rosuvastatin (C10AA07)	5.8 %
Gemfibrozil (C10AB04) to Atorvastatin (C10AA05)	5.6 %
Atorvastatin (C10AA05) to Gemfibrozil (C10AB04)	5.6 %
Other combinations	<5.0 % each

Within our sample, we found that the average DDD of statin therapy gradually increased over time from about 1.03 DDD at the start to about 1.3 DDD at the end of follow-up (ca. 5000 days later).

### Statin adherence

**Fig. 1:**
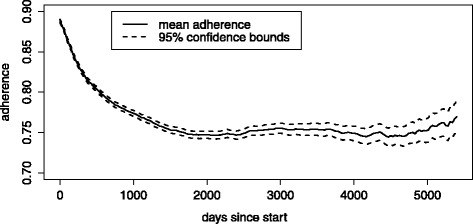
Adherence over time among individuals aged 46 to 100 in the period 1996 to 2012

Average adherence to statin therapy decreased strongly in the first 1000 days of follow up, until approximately 74 % adherence. Adherence then remained approximately constant over time (Fig. 1). For the majority of observations, individual patients were either highly adherent (adherence ≥ 0.95) or highly non-adherent (adherence ≤ 0.05). The percentage fully adherent appeared to remain stable, while the percentage non-adherent increased over time.

### Mortality

During the study period from 1996 to 2010, of the 49,688 patients in the sample, 1033 died due to ischemic heart disease and 532 due to cerebrovascular disease, which together form the category ‘cardiovascular mortality’. Non-cardiovascular causes were responsible for 6594 deaths among these patients. Among these non-cardiovascular causes, 1179 died from the falsification end-point.

### Small Cox model

In a model including statin adherence, statin exposure level, age and age squared as continuous variables, and period as categorical variable, the conditional estimate was that being fully adherent reduced the hazard of cardiovascular mortality by about 30 % (HR: 0.70, 95 % CI: 0.61 to 0.81) compared to being fully non-adherent (Table 3). Birth cohort did not add significantly to this model (*p* = 0.51), and its addition did not change the parameter estimate of statin adherence. The interaction term between statin adherence and birth cohort also did not add significantly to the model (*p* = 0.81).

**Table 3:** Effect estimates of adherence to statin therapy on cardiovascular mortality by calendar period

Calendar years	Small model		Large model		Difference in HR	# events
	HR	95 % CI	HR	95 % CI		
1994–2002	0.85	0.55 to 1.34	0.79	0.44 to 1.40	0.06	158
2003–2006	0.58	0.45 to 0.76	0.46	0.35 to 0.59	0.12	642
2007–2012	0.77	0.65 to 0.92	0.54	0.45 to 0.65	0.23	765
Overall (1994–2002)	0.70	0.61 to 0.81	0.53	0.46 to 0.61	0.17	1565
Falsification outcome (1994–2002)	0.93	0.97 to 1.09	0.68	0.58 to 0.80	0.25	1179

### Large Cox model

The final model included the following variables as continuous variables: statin adherence and exposure level, age and age squared, diuretic adherence and exposure level, and obstructive airway drug adherence and exposure level. A number of other variables were added as categorical variables: calendar year, sex, anti-thrombotic agent adherence and exposure level, anti-inflammatory & anti-rheumatic drug adherence and exposure level, cardiac therapy adherence and exposure level, beta-blocking agent adherence and exposure level, and calcium channel blocker adherence and exposure level. Birth cohort did not add significantly to this model (*p* = 0.61), and adding birth cohort did not have a strong effect on the effect estimates of statin adherence. Socio-economic status also did not add significantly to the model (*p* = 0.83). The final model did not include the diabetes treatment variable because the effect estimate of statin adherence did not change more than 5 % after adjustment for diabetes treatment. The conditional estimate was that being fully adherent to statins reduced the hazard of cardiovascular mortality by about 47 % (HR: 0.53; 95 % CI: 0.46 to 0.61) (Table 3). This estimate was similar to the population-averaged estimate; using the parametric G-formula, in the scenario where all patients were fully adherent, the hazard was reduced by 49 % (HR: 0.51; 95 % CI 0.43 to 0.61). Including birth cohort in the G-formula did not change this population-averaged estimate substantially (HR: 0.50, 95 % CI 0.42 to 0.59).

### Falsification end-points

Being adherent to statin therapy was not protective against respiratory, endocrine, nutritional and metabolic disease in the small model (HR: 0.93, 95 % CI: 0.79 to 1.09), which argues against the presence healthy adherer bias (Table 3). However, in the large model it did appear to be protective (HR: 0.68, 95 % CI: 0.58 to 0.80). Separate analyses for mortality due to respiratory diseases, and mortality due to endocrine, nutritional and metabolic diseases yielded results of similar magnitude.

### Subset analyses

The estimated effect of adherence to statin therapy on the hazard of cardiovascular mortality changed over time (Table 3). The difference between the estimates of the large and the small models, as an indication of measured confounding, was larger in more recent years.

## Discussion

For the population aged 46 to 100 years in the study period 1996 to 2012 in the Netherlands, both the conditional effect estimate and the population averaged effect estimate indicated that being adherent to statin therapy was strongly protective against cardiovascular mortality. Including birth cohort or neighborhood SES covariates did not affect the estimate of the effect of the primary exposure on the primary outcome. The differences between the estimates from the small and the large Cox models substantially changed between time periods. Furthermore, in the simple model, the falsification end-point did not indicate bias, but in the large model there was a strong indication of bias. Confounding also appeared to differ by calendar period.

### Falsification end-points and sources of bias

In this study, we avoided confounding by indication by comparing statin therapy starters amongst each other. We investigated whether healthy adherer bias affected our results by also analyzing the effect of statin adherence on falsification outcomes. In the small model, statin adherence was not protective against falsification outcomes, which means there is no indication of healthy adherer bias. However, in the large model, statin adherence did become protective against the falsification outcomes, while also becoming protective against cardiovascular mortality. A criticism of the falsification end-point approach is that the falsification end-point and the primary end-point are not necessarily affected by the same bias [9]. However, the effect of statin adherence on both outcomes became biased in the same direction, and the relative magnitude of the bias was also the same (0.70/0.53 = 1.32 for CVD and 0.93/0.68 = 1.36 for the falsification outcomes). These indicate that both outcomes were likely affected by the same bias.

Some causal pathways from statin utilization to respiratory, endocrine and metabolic mortality are possible. We believe these pathways to be limited, and therefore to only have a minor effect on the association between statin utilization and the falsification outcomes. Nevertheless, ideally, we would have chosen deaths due to external causes (e.g. deaths as a result of accidents and physical abuse) as falsification end-points, as they are even less likely to be affected by lipid levels than the current choice. However, only ~100 events due to this cause were recorded, and hence this choice is underpowered.

Since healthy adherer bias appears to be limited, and the bias is caused by adjusting for an increased set of covariates, the source of the bias is likely either overadjustment or competing risks [25, 26]. Overadjustment bias can be caused by conditioning on mediators or on colliders [25]. However, none of the added variables should mediate the effect of statin adherence on cardiovascular mortality (or the falsification end-points), and none should function as colliders in this context. The bias is therefore likely caused by competing risks. Next to cardiovascular mortality (and the falsification outcomes), patients may die from a large number of other causes of death. By fitting a Cox model to data in which competing risks are present, we model the cause-specific hazard. Cause specific hazards are the hazards at time *t* of a specific cause of death conditional on surviving to time *t*. That is, conditional on not having died from the event under study before time *t*, as well as not having died from a competing event before time *t*. Therefore, the hazards of the competing cause of death affect the hazard of cardiovascular mortality. If the additional covariates in the large Cox model in this study affect the hazards of competing risks, then this also affects the hazard of cardiovascular mortality and the falsification end-points. This problem would not arise if we could model the marginal cause-specific hazard, i.e. the hazard of cardiovascular mortality where the hazards of competing causes are 0. However, the marginal cause-specific hazards are unfortunately unobservable.

We also compared bias between different calendar periods, and observed that the difference between the effect estimates of the small and the large models increased over calendar time. Overall, in the large model, the effect estimates of statin adherence on cardiovascular mortality were closer to that of clinical trials in the period prior to 2002. In the period prior to the year 2002, statins were especially indicated for patients between ages 50 to 70 years with hypercholesterolemia [1]. Around the year 2002, important studies showed that also patients above age 70, and that diabetic patients, benefitted from statins. In the Netherlands in the year 2006, the age restrictions were formally abolished. Therefore, the patient population likely resembled the trial population more closely shortly after the introduction of statins in the population, and hence effect estimates are also more similar. Furthermore, it is possible that due to the studies and guideline changes, the patient population became more heterogeneous over time, and adjustment for potential confounders more strongly changed the effect estimate of statin therapy.

### Parametric G-formula

Because Cox models are non-collapsible [22], we used the parametric G-formula (a method of direct standardization) to produce a population averaged effect estimate for the effect of statin therapy on the hazard of cardiovascular mortality. The parametric G-formula is only rarely employed [24], but can be highly useful, as it shows the effect of a time-dependent intervention on the population level. In our study, the population-averaged estimate shows the effect on the hazard if all statin users in the population were fully adherent at all times from first dispensing onwards, compared to the situation where they were all fully non-adherent at all times. The population averaged estimate is close to the conditional effect estimate, which is likely caused by the low hazard of cardiovascular mortality (at any time point) in our sample. For this reason, we chose not to apply the parametric G-formula in subset analyses.

### Birth cohort and confounding & effect modification

In this study, we conclude that non-linear birth cohort does not confound the estimates of the effect of statin adherence on the hazard of cardiovascular mortality. It may still be possible that the linear part of birth cohort confounds the outcome, however this is less problematic because age and calendar time are commonly included in analyses of drug effectiveness, and would therefore also include linear birth cohort through the dependency between the three variables [21]. In this way, it may even be possible to model away a true birth cohort effect by using non-linear terms (including interaction effects) for age and period.

If birth cohort is (conditional on age and calendar year) related to health behavior, then this would be relevant for two reasons. First, birth cohort may affect healthy adherer bias and therefore controlling for birth cohort should result in more valid estimates of the causal effect of statin adherence on cardiovascular mortality when information on health behavior itself is unavailable. However, since we did not find evidence for confounding by birth cohort, it is less likely that birth cohort is strongly related to health behavior on the patient level. Secondly, it may mean that the effectiveness of drugs is different for different birth cohorts, because cohorts would have differences in the way they utilize drugs. Since we did not find evidence for effect modification by birth cohort, this also appears to be less likely. It could be argued that since adherence to statins and other drugs is itself an indicator of health behavior, looking at statin adherence (and adherence of other drugs) removes the effect of birth cohort on cardiovascular mortality.

### Statin therapy effectiveness

Being adherent to statins appears to be protective against cardiovascular mortality, which is in line with the literature, though clinical effect may differ between subgroups (e.g. [27-29]). We shall here interpret the results from the small Cox model, as the larger model is known to be biased. In the small model, the population-averaged hazard ratio of statin adherence was estimated to be 0.70 (95 % CI: 0.61 to 0.81). This means that the hazard of cardiovascular mortality of a patient who is fully adherent is 30 % lower than the hazard of cardiovascular mortality in a similar patient who is completely non-adherent. This estimate is close to that of a Cochrane review of randomized clinical trials, but the confidence intervals do not overlap. In the Cochrane review, the hazard was estimated to be reduced by 17 % against fatal cardiovascular events (RR: 0.83, 95 % CI: 0.72 to 0.96). Differences may be caused by our study population being a relatively low-risk population for cardiovascular mortality (1565/49,688 * 100 % ≈ 3 % probability of CVD death).

### Evaluation of data and methods

The findings of the study in regard to statin effectiveness are not directly comparable with those of earlier observational studies because the outcome definitions differed, as well as the definition of the primary exposure. To the best of our knowledge, this is the first study that used time-varying adherence to statin therapy as the primary exposure. Other studies that have related statin adherence to cardiovascular outcomes commonly calculate adherence over a fixed period, such as adherence in the first year. Using adherence in the first year is useful for predictive (and therefore clinical) purposes. However, time invariant adherence will likely be less strongly related to the outcome; a patient’s adherence in the first year should not be strongly related to his or her adherence in the 5^th^ year of follow up, and therefore to the hazard of mortality in the fifth or sixth year. This shows the usefulness of accounting for time-varying drug adherence [12].

The proportion of days covered method is a method of indirect observation. It’s strength, compared to direct methods, is that direct methods are intensive and invasive, which therefore often severely limit the number of observations that can be performed [12]. However, a limitation of indirect methods is that it cannot be observed if patients take their statin [12]. This overestimates adherence and thereby biases statin effect estimates towards the null. However, this bias may be limited with the proportion of days covered method, given that patients who forget to take their statin are less quickly in need of new statins and therefore their adherence estimate is adjusted downwards.

More than 90 % of the patients that were censored in the study were subject to administrative censoring, which is non-informative. That is, they were still being followed when the study ended on the 31^st^ of December 2012. The remaining number of patients were censored during the study: if this did not occur due to competing mortality, it could only occur due to patients moving out of the IADB coverage area due to the type of data sources that were used. It is unknown to what extent a move is related to impending cardiovascular mortality.

Due to data limitations, we were unable to determine the underlying health conditions of individual patients (except by using drugs dispensed as proxies for these conditions). Therefore, we could not determine which patients had familial hypercholesterolemia. However, the prevalence of this illness in the Netherlands is ~0.25 %, and therefore is unlikely to have strongly biased our estimates [30].

## Conclusions

In time-to-event analysis in a competing risks setting, adjusting for confounding, while necessary, can cause new biases to emerge. Falsification end-points can help detect this bias, and is therefore a useful approach in such a complex setting. However, this study generates evidence that for the population aged 46 to 100 in the study period 1996 to 2012 in the Netherlands, being adherent to statin therapy appeared to lower the risk of cardiovascular mortality, compared to being not adherent.

## Ethics approval and consent to participate

Permission from an institutional review board was not required to perform this study.
